# Concurrent Chemoradiation for Vaginal Cancer

**DOI:** 10.1371/journal.pone.0065048

**Published:** 2013-06-07

**Authors:** David T. Miyamoto, Akila N. Viswanathan

**Affiliations:** 1 Harvard Radiation Oncology Program, Harvard Medical School, Boston, Massachusetts, United States of America; 2 Department of Radiation Oncology, Dana-Farber Cancer Institute/Brigham & Women’s Hospital, Boston, Massachusetts, United States of America; National University of Ireland Galway, Ireland

## Abstract

**Background:**

It is not known whether the addition of chemotherapy to radiation therapy improves outcomes in primary vaginal cancer. Here, we review clinical outcomes in patients with primary vaginal cancer treated with radiation therapy (RT) or concurrent chemoradiation therapy (CRT).

**Methods:**

Seventy-one patients with primary vaginal cancer treated with definitive RT with or without concurrent chemotherapy at a single institution were identified and their records reviewed. A total of 51 patients were treated with RT alone; 20 patients were treated with CRT. Recurrences were analyzed. Overall survival (OS) and disease-free survival (DFS) rates were estimated using the Kaplan-Meier method. Cox regression analysis was performed.

**Results:**

The median age at diagnosis was 61 years (range, 18–92 years) and the median follow-up time among survivors was 3.0 years. Kaplan-Meier estimates for OS and DFS differed significantly between the RT and CRT groups (3-yr OS = 56% vs. 79%, log-rank *p = *0.037; 3-yr DFS = 43% vs. 73%, log-rank *p = *0.011). Twenty-three patients (45%) in the RT group had a relapse at any site compared to 3 (15%) in the CRT group (*p = *0.027). With regard to the sites of first relapse, 10 patients (14%) had local only, 4 (6%) had local and regional, 9 (13%) had regional only, 1 (1%) had regional and distant, and 2 (3%) had distant only relapse. On univariate analysis, the use of concurrent chemotherapy, FIGO stage, tumor size, and date of diagnosis were significant predictors of DFS. On multivariate analysis, the use of concurrent chemotherapy remained a significant predictor of DFS (hazard ratio 0.31 (95% CI, 0.10–0.97; p = 0.04)).

**Conclusions:**

Vaginal cancer results in poor outcomes. Adequate radiation dose is essential to ensure curative management. Concurrent chemotherapy should be considered for vaginal cancer patients.

## Introduction

Vaginal cancer is a rare disease, comprising only 1%–2% of all gynecologic malignancies. Known prognostic factors for recurrence of vaginal cancer include stage, lymph node involvement [1], [2], size of the initial lesion [3] and advanced age [4]. Location of the lesion, grade, and HPV status have conflicting evidence [5].

The standard treatment for non-metastatic vaginal cancer has historically consisted of definitive radiation therapy (RT) alone, using external-beam radiation therapy (EBRT) with or without brachytherapy [6], [7], [8]. Optimal outcomes are achieved with careful selection of treatment modalities to ensure coverage of the tumor and involved regions. Over the past two decades, the integration of computed tomography (CT) and magnetic resonance imaging (MRI) for staging has allowed more accurate assessment of nodal involvement and appropriate treatment to suspicious lymph node regions. Similarly, 3-dimensional (3D) imaging used for radiation planning has facilitated the identification and delineation of the target volume with greater accuracy [9].

Given the favorable outcome of cervical cancer patients treated with chemoradiation therapy (CRT) [10], [11], translation to vaginal cancer remains a topic of discussion. Studies published to date address the feasibility and outcomes of CRT for primary vaginal cancer, but have few patients and therefore comparisons to RT alone are limited [12], [13], [14]. In this study, we analyze and compare the clinical outcomes of patients with primary vaginal cancer treated at our institution with either definitive RT or with CRT.

## Patients and Methods

### Patients

Records of patients with a diagnosis of biopsy-proven primary vaginal cancer treated with definitive RT with or without concurrent chemotherapy at Brigham & Women’s Hospital/Dana-Farber Cancer Institute between 1972 and 2009 were identified and retrospectively reviewed under an Institutional Review Board (Dana-Farber Cancer Institute IRB)-approved protocol with a waiver of consent. Exclusion criteria were prior gynecological malignancy, prior pelvic radiation therapy, metastatic disease and cancer involvement of the cervix or vulva. For all patients, pretreatment patient and tumor characteristics were recorded, including date of diagnosis, stage according to the International Federation of Gynecology and Obstetrics (FIGO) 2000 classification, histology, tumor size, tumor site, tumor differentiation, presence of lymphatic vessel invasion (LVI) and lymph node involvement. For each patient, medical records were reviewed to determine whether diagnostic imaging, including pelvic CT, pelvic MRI and PET scan, had been performed to evaluate for lymph node involvement and/or distant metastases.

### Treatment

Treatment characteristics for all patients were recorded, including the type of radiation therapy used (EBRT, brachytherapy, or both), EBRT technique, brachytherapy type and dose rate, treatment duration, and the use of chemotherapy. For patients treated with high-dose-rate (HDR) brachytherapy, the total radiation dose was converted to the biologically equivalent dose (BED) in 2-Gy fractions, using the linear quadratic BED equation with an α/β ratio of 10. For patients treated with CRT, the type of chemotherapy used and the number of cycles of chemotherapy were recorded. Information was also collected on whether surgery was performed and the type of surgery.

### Clinical Outcomes

Data on outcomes were collected for each patient, including local, regional and distant relapse, disease-free survival (DFS) and overall survival (OS). *Local relapse* was defined as recurrence in the vagina or paravaginal area. *Regional relapse* was defined as recurrence in the pelvic or inguinal lymph nodes. *Distant relapse* included para-aortic recurrences and distant metastases.

### Statistical Analysis

We report medians with interquartile range or means with standard deviation for numeric variables and percentages for categorical or ordinal variables. Treatment groups were compared using the exact Wilcoxon test for numeric or ordinal variables, the Fisher exact test for binary variables, and the likelihood ratio test for multicategory discrete variables. Two-sided *p*-values <0.05 were considered statistically significant. OS was measured from the date of the start of RT to the date of death or last follow-up. Survival curves were generated using the Kaplan-Meier method and compared using the log-rank test. Univariate and multivariate Cox regression analyses were used to identify predictors of OS and DFS. Statistical analyses were performed using R, version 2.12.0.

## Results

We identified 71 patients with primary vaginal cancer treated with definitive RT with or without concurrent chemotherapy at Brigham & Women’s Hospital/Dana-Farber Cancer Institute between 1972 and 2009. A total of 51 patients were treated with RT without chemotherapy and 20 were treated with CRT.

### Patient and Tumor Characteristics

Detailed patient and tumor characteristics are listed in Table 1. The median age was 61 years for all patients. There were no significant differences between the RT-alone and CRT groups with regard to FIGO stage, tumor size, histology, differentiation or presence of LVI. There was a statistically significant difference in terms of the year of diagnosis; more patients treated with RT alone were diagnosed prior to 1999, and more patients treated with CRT were diagnosed after 1999. With regard to tumor site, there was a trend towards a higher proportion of patients in the CRT group having involvement of the lower 1/3 of the vagina. A significantly higher percentage of patients in the CRT group than the RT-alone group underwent diagnostic imaging; also, significantly more patients in that cohort had radiographically detected lymph node involvement.

**Table 1:** Patient and tumor characteristics.

Category	Subcategory	All patients (n = 71)	RT alone (n = 51)	ChemoRT (n = 20)	*p* value
Median follow-up (range)	All patients	2.3 years (0.3–14.6)	2.3 years (0.5–14.6)	2.6 years (0.3–7.76)	0.625
	Survivors	3.0 years (0.3–14.6)	3.0 years (0.7–14.6)	3.6 years (0.3–7.8)	0.462
Median Age (range)	All patients	61 (18–92)	64 (20–92)	60 (18–81)	0.112
Year of diagnosis	1970–1979	8 (11%)	8 (16%)	0 (0%)	**<0.001**
	1980–1989	14 (20%)	13 (26%)	1 (5%)	
	1990–1999	17 (24%)	16 (32%)	1 (5%)	
	2000–2009	32 (45%)	14 (28%)	18 (90%)	
Stage (FIGO)	I	22 (31%)	18 (35%)	4 (20%)	0.395
	II	30 (42%)	19 (37%)	11 (55%)	
	III	12 (17%)	8 (16%)	4 (20%)	
	IVA	7 (10%)	6 (12%)	1 (5%)	
Prior hysterectomy	No	33 (47%)	21 (41%)	12 (60%)	0.191
	Yes	38 (54%)	30 (59%)	8 (40%)	
Diagnostic imaging	No	30 (42%)	29 (57%)	1 (5%)	**<0.001**
	Yes	41 (58%)	22 (43%)	19 (95%)	
	CT	21 (30%)	16 (31%)	5 (25%)	
	MRI	9 (13%)	2 (4%)	7 (35%)	
	PET	1 (1%)	1 (2%)	0 (0%)	
	CT and MRI	4 (6%)	1 (2%)	3 (15%)	
	MRI and PET	5 (7%)	1 (2%)	4 (20%)	
	CT and PET	1 (1%)	1 (2%)	0 (0%)	
Tumor size	≤4 cm	41 (58%)	27 (53%)	14 (70%)	0.202
	>4 cm	24 (34%)	18 (35%)	6 (30%)	
	Unknown	6 (9%)	6 (12%)	0 (0%)	
Tumor site	Apex	15 (21%)	12 (24%)	3 (15%)	0.055
	Upper 2/3	39 (55%)	31 (61%)	8 (40%)	
	Lower 1/3	10 (14%)	4 (8%)	6 (30%)	
	Entire	6 (9%)	4 (8%)	2 (10%)	
	Unknown	1 (1%)	0 (0%)	1 (5%)	
Histology	Squamous cell	54 (76%)	36 (71%)	18 (90%)	0.384
	Adenocarcinoma	7 (10%)	7 (14%)	0 (0%)	
	Clear cell	5 (7%)	4 (8%)	1 (5%)	
	Epidermoid	2 (3%)	2 (4%)	0 (0%)	
	Unknown	2 (3%)	1 (2%)	1 (5%)	
	Other	1 (1%)	1 (2%)	0 (0%)	
Differentiation	Well	10 (14%)	9 (18%)	1 (5%)	0.116
	Moderately	18 (25%)	15 (29%)	3 (15%)	
	Poorly	25 (35%)	14 (28%)	11 (55%)	
	Unknown	18 (25%)	13 (26%)	5 (25%)	
LVI	No	10 (14%)	7 (14%)	3 (15%)	0.923
	Yes	5 (7%)	3 (6%)	2 (10%)	
	Unknown	56 (79%)	41 (80%)	15 (75%)	
Lymph node involvement	None	61 (86%)	48 (94%)	13 (65%)	**0.004**
	Pelvic	4 (6%)	2 (4%)	2 (10%)	
	Inguinal	2 (3%)	0 (0%)	2 (10%)	
	Pelvic and inguinal	1 (1%)	1 (2%)	0 (0%)	
	Para-aortic	3 (4%)	0 (0%)	3 (15%)	

Key: CRT, concurrent chemoradiation; FIGO, Federation Internationale de Gynecologie et Obstetrique; CT, computed tomography; MRI, magnetic resonance imaging; PET, positron emission tomography; LVI, lymphatic vessel invasion.

### Treatment Details

Treatment characteristics for all patients are listed in Table 2. In the RT-alone group, most patients were treated with EBRT and brachytherapy, although some patients received EBRT alone or brachytherapy alone. In the CRT group, all patients were treated with both EBRT and brachytherapy. Twenty-eight percent of all patients underwent surgery as part of their initial management prior to RT or CRT, including 35% of patients in the RT-only group and 10% of those in the CRT group. Most of these patients underwent either a vaginectomy (16%) or a wide local excision of tumor (10%).

**Table 2:** Treatment characteristics.

Category	Subcategory	All patients (n = 71)	RT alone (n = 51)	ChemoRT (n = 20)	*p* value
Radiation therapy given	Brachytherapy alone	9 (13%)	9 (18%)	0 (0%)	**0.006**
	EBRT alone	10 (14%)	10 (20%)	0 (0%)	
	EBRT+brachytherapy	52 (73%)	32 (63%)	20 (100%)	
RT dose to Vagina (EQD2)	All RT groups	68.2±15.4 Gy	65.1±16.8 Gy	76.0±6.5 Gy	0.99
	Brachy alone group	54.2±22.2 Gy	54.2±22.2 Gy	–	–
	EBRT alone group	48.4±7.1 Gy	48.4±7.1 Gy	–	–
	EBRT+brachy group	74.4±9.3 Gy	73.5±10.7 Gy	76.0±6.5 Gy	0.90
EBRT fields	4-field	25 (35%)	18 (35%)	7 (35%)	0.654
	AP/PA	32 (45%)	21 (41%)	11 (55%)	
	Opposed laterals	1 (1%)	1 (2%)	0 (0%)	
	3-field	1 (1%)	0 (0%)	1 (5%)	
	En face	1 (1%)	1 (2%)	0 (0%)	
	Unknown	2 (3%)	1 (2%)	1 (5%)	
Brachytherapy type	Cylinder	26 (37%)	21 (41%)	5 (25%)	0.235
	Interstitial	26 (37%)	16 (31%)	10 (50%)	
	Tandem and ovoid	4 (6%)	1 (2%)	3 (15%)	
	Cylinder and tandem	2 (3%)	1 (2%)	1 (5%)	
	Interstitial and tandem	2 (3%)	1 (2%)	1 (5%)	
	Sutures	1 (1%)	1 (2%)	0 (0%)	
Brachytherapy dose rate	HDR	23 (32%)	12 (24%)	11 (55%)	0.090
	LDR	38 (54%)	29 (57%)	9 (45%)	
Concurrent Chemotherapy	Yes	20 (28%)	–	–	–
	Cisplatin	17 (24%)	–	17 (85%)	
	Carboplatin	2 (3%)	–	2 (10%)	
	5-FU	1 (1%)	–	1 (5%)	
	Unknown	0 (1%)	–	0 (0%)	
Number of cycles chemo	ChemoRT group	5 (1–8)	–	5 (1–8)	–
Surgery	Yes	20 (28%)	18 (35%)	2 (10%)	**0.040**
	Wide excision	7 (10%)	6 (12%)	1 (5%)	
	Vaginectomy	11 (16%)	10 (20%)	1 (5%)	
	Pelvic exenteration	2 (3%)	2 (4%)	0 (0%)	

Key: CRT, concurrent chemoradiation; EBRT, external-beam radiation therapy; EQD2, Equivalent dose in 2Gy fractions; AP/PA, anterioposterior/posterioanterior; HDR, high-dose-rate; LDR, low-dose-rate.

The mean total radiation dose received for all patients, calculated as the BED in 2-Gy fractions, was 68.2±15.4 Gy. The difference in radiation dose received between patients in the RT-alone group and those treated with CRT was not statistically significant (Table 2). The subgroup of patients treated with both EBRT and brachytherapy received a higher total dose of radiation (BED, 74.4±9.3 Gy) than those treated with EBRT alone; however, this also did not significantly differ between the RT-alone and CRT groups. The technique and field arrangements of EBRT are described in Table 2. There were no significant differences in technique between the RT-alone and CRT groups. Most patients received EBRT with a 4-field approach or anterioposterior/posterioanterior fields. With regard to brachytherapy technique, there was no significant difference in the types of brachytherapy applicators used between the two groups. However there was a trend towards a higher percentage of patients in the CRT group receiving HDR brachytherapy as opposed to low-dose-rate (LDR) brachytherapy.

Among the CRT patients, 85% received weekly cisplatin concurrently with RT at a dose of 40 mg/m^2^; the remainder received either carboplatin or 5-FU during RT. The average number of cycles of concurrent chemotherapy completed was five. One patient received adjuvant doxorubicin and paclitaxel after completion of CRT with concurrent cisplatin. Another patient received neoadjuvant cisplatin and 5-FU prior to undergoing CRT with concurrent 5-FU. One patient who was included in the RT-only group received carboplatin and cyclophosphamide prior to her radiation.

### Outcome

**Figure 1:**
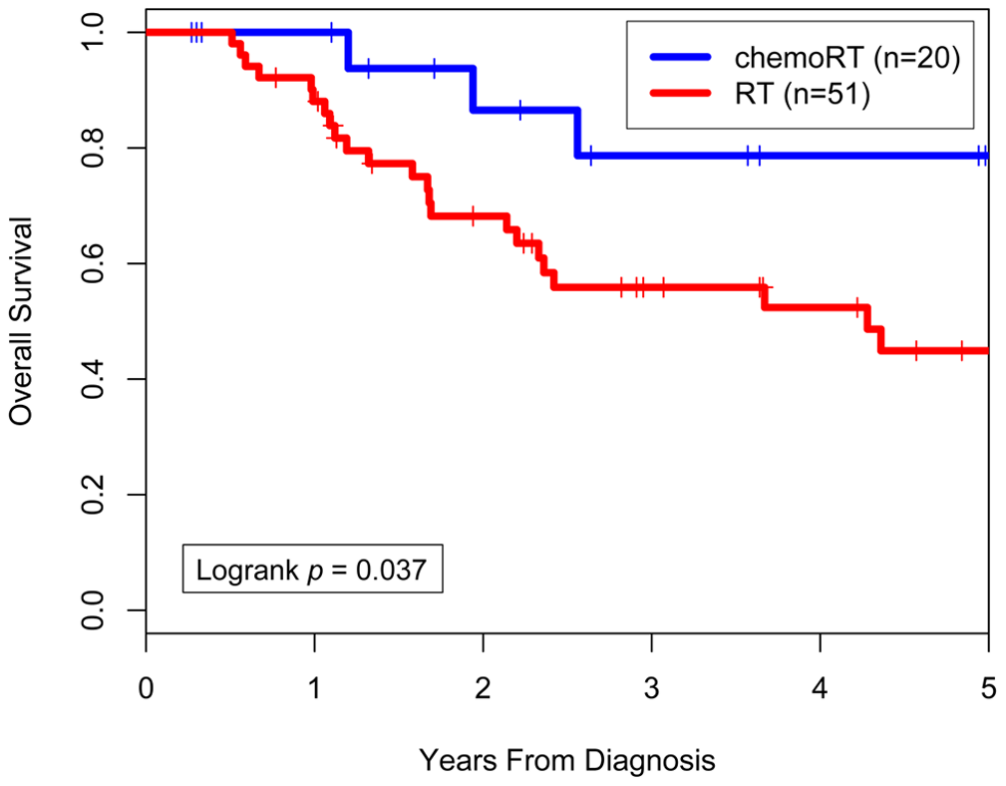
Overall survival by treatment (chemoradiation therapy or radiation therapy alone).

**Figure 2:**
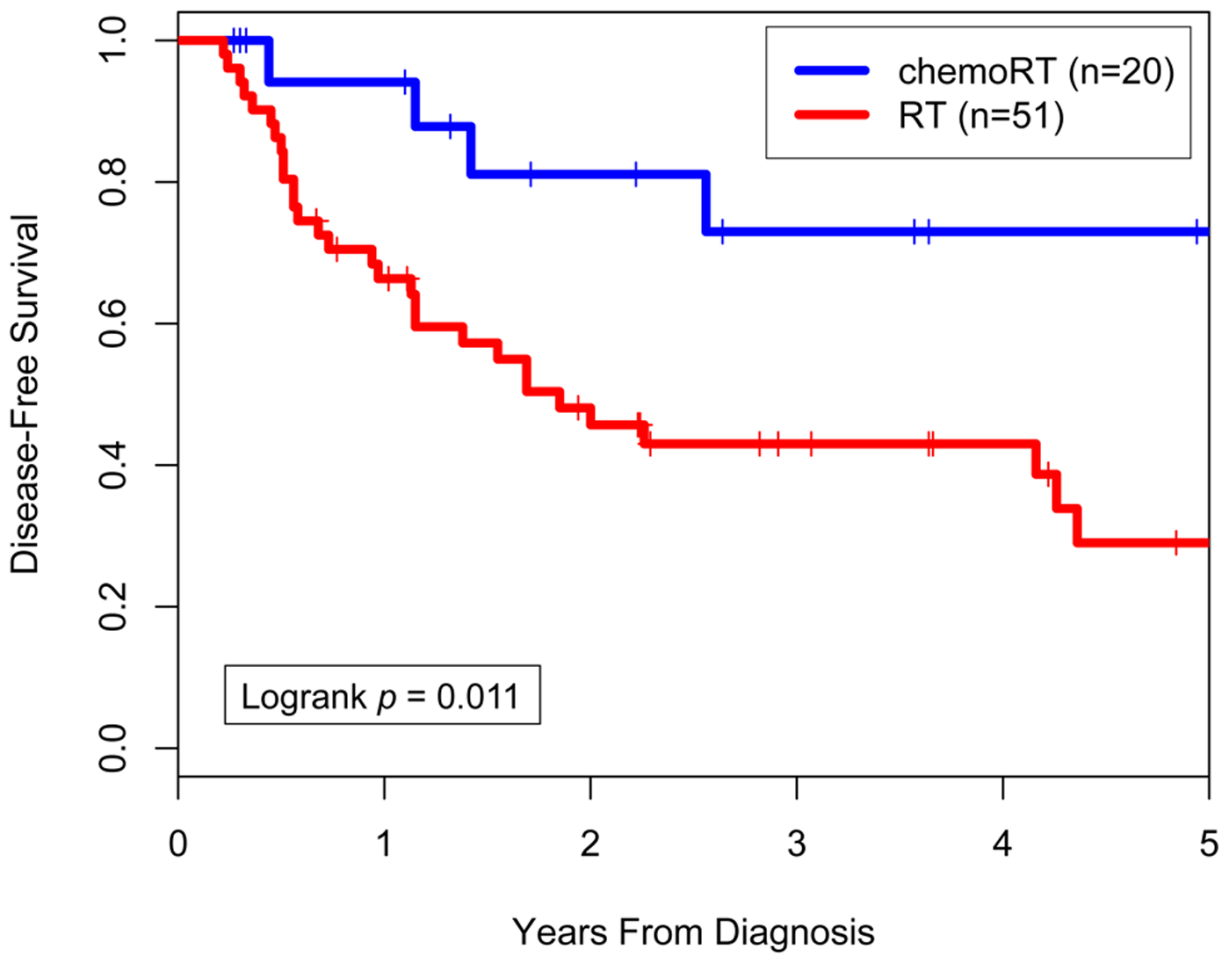
Disease-free survival by treatment (chemoradiation therapy or radiation therapy alone).

The median follow-up time among survivors was 3.0 years for all patients. Median follow-up time was not significantly different between the RT-alone and CRT groups (Table 1). Kaplan-Meier actuarial OS rates at 3 years were 56% for the RT-alone group and 79% for the CRT group (log-rank *p* = 0.037; Figure 1). Median OS time was 4.3 years for the RT-alone group and had not been reached for the CRT group. DFS was also significantly different between the two groups, with actuarial 3-year DFS rates of 43% for the RT-alone group and 73% for the CRT group (log-rank *p* = 0.011; Figure 2). Median DFS time was 1.9 years for the RT-alone group and had not been reached for the CRT group.

A subgroup analysis was performed for the 52 patients who received both EBRT and brachytherapy; 32 of these were in the RT-alone group and 20 in the CRT group. Within this subgroup, actuarial 3-year DFS rates remained significantly different between the RT-alone and CRT groups (48% vs. 73%; log-rank *p* = 0.027) and actuarial 3-year OS trended towards a difference favoring the CRT group (62% vs. 79%; log-rank *p* = 0.113).

### Patterns of Tumor Relapse

**Figure 3:**
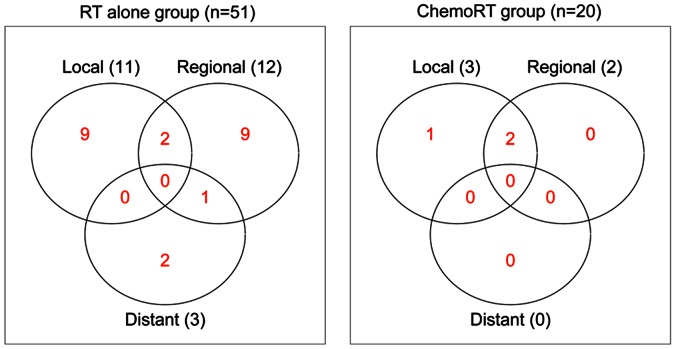
Sites of first relapse by treatment group (radiation therapy alone compared to chemoradiation therapy).

A total of 26 patients (37%) had a relapse of vaginal cancer after treatment. With regard to the sites of first relapse, 10 patients (14%) had local only, 4 (6%) had local and regional, 9 (13%) had regional only, 1 (1%) had regional and distant, and 2 (3%) had distant only relapse. The percentage of patients with relapse was significantly higher in the RT-alone group (n = 23, 45%) than in the CRT group (n = 3, 15%; *p* = 0.027). The distribution of the first sites of vaginal-cancer relapse is depicted in graphical form in Figure 3.

### Cox Proportional Hazards Analyses

Results from univariate Cox proportional hazards analyses for DFS and OS are presented in Table 3. On univariate analysis for predictors of DFS, year of diagnosis, use of concurrent chemotherapy, FIGO stage, and tumor size >4 cm were significant variables. Variables that were not significant included lymph node involvement, tumor differentiation, age at diagnosis, use of surgery, prior hysterectomy, treatment duration, and use of 3D diagnostic imaging. Significant univariate predictors of OS included year of diagnosis, use of concurrent chemotherapy, radiation dose (EQD2), use of both EBRT and brachytherapy, and any use of brachytherapy. Radiation dose was significant both as a continuous variable and when dichotomized by the median dose of 70 Gy. Lymph node involvement, tumor differentiation, age at diagnosis, use of surgery, prior hysterectomy, treatment duration, use of 3D diagnostic imaging, FIGO stage, and tumor size were not significant univariate predictors of OS.

**Table 3:** Cox proportional hazards analyses.

Univariate	Disease-Free Survival		Overall Survival	
Variable	Crude HR (95% CI)	P value (Wald)	Crude HR (95% CI)	P value (Wald)
Date of diagnosis (year)	0.96 (0.93–0.99)	**0.011**	0.96 (0.93–0.99)	**0.030**
Chemotherapy	0.28 (0.10–0.80)	**0.017**	0.30 (0.09–0.99)	**0.049**
FIGO stage	1.53 (1.08–2.16)	**0.017**	1.38 (0.95–2.00)	0.096
Tumor size >4 cm	2.12 (1.04–4.35)	**0.040**	1.84 (0.83–4.06)	0.131
Treatment duration (days)	0.99 (0.97–1.001)	0.072	0.98 (0.97–1.001)	0.062
3D Diagnostic imaging	0.54 (0.28–1.07)	0.077	0.67 (0.31–1.42)	0.295
Brachytherapy (any)	0.50 (0.22–1.10)	0.084	0.42 (0.18–0.97)	**0.041**
RT dose to vagina (>70 Gy EQD2)	0.55 (0.27–1.09)	0.085	0.34 (0.15–0.77)	**0.009**
Age at diagnosis	1.02 (0.99–1.04)	0.096	1.02 (0.999–1.04)	0.066
Prior hysterectomy	1.80 (0.89–3.62)	0.100	1.71 (0.81–3.60)	0.156
Tumor differentiation	0.71 (0.45–1.11)	0.130	0.65 (0.39–1.11)	0.107
EBRT and brachytherapy	0.59 (0.30–1.18)	0.138	0.44 (0.20–0.94)	**0.034**
Lymph node involvement	0.90 (0.35–2.32)	0.822	0.86 (0.30–2.49)	0.781
Surgery	0.99 (0.49–2.00)	0.980	0.93 (0.42–2.07)	0.866

Key: HR, hazard ratio; FIGO, Federation Internationale de Gynecologie et Obstetrique; EQD2, Equivalent dose in 2Gy fractions; EBRT, external-beam radiation therapy.

Given the total of 26 relapse events, the number of variables that could be included in the multivariate model was limited. We therefore tested several clinically relevant variables in combination, realizing that the final multivariate model would be weakened by the small number of events. We included year of diagnosis in the models to attempt to control for changes over time. On including tumor size in a model for DFS that included the use of concurrent chemotherapy, FIGO stage, and year of diagnosis, tumor size was no longer significant, and the HR for chemotherapy remained significant at 0.31 (95% CI 0.10–0.98; p = 0.05). To assess the impact of 3D diagnostic imaging, this variable was evaluated in the multivariate model for DFS. With this exploratory analysis, the HR for the use of concurrent chemotherapy remained significant at 0.31 (95% CI 0.10–0.97; p = 0.04). However, the use of diagnostic imaging itself was not significant on univariate analysis (HR 0.54; 95% CI, 0.28–1.07; *p* = 0.08) and was therefore not included in the final model. Similarly, total RT dose to the vagina (≥70 Gy EQD2) was not a significant predictor for DFS on MVA. Therefore, our final multivariate model for DFS included the use of chemotherapy, FIGO stage, and year of diagnosis, with which the DFS hazard ratio for the use of concurrent chemotherapy remained significant at 0.31 (95% CI, 0.10–0.97; p = 0.04).

## Discussion

This retrospective single-institution study demonstrates a potential benefit in overall survival and disease-free survival with the addition of concurrent chemotherapy in the treatment of primary vaginal cancer. In addition to the use of concurrent chemotherapy, other significant covariates in univariate models for disease-free and overall survival included radiation dose, FIGO stage, tumor size and year of diagnosis. Multivariate analysis was limited given the small number of events. Whether concurrent chemotherapy contributes to a long-term overall survival benefit in patients who receive sufficient radiation in the modern era must be tested in a larger study.

Prospective randomized controlled trials have demonstrated a survival benefit with the addition of concurrent chemotherapy to RT in the treatment of cervical cancer [10], [11]. Other studies have demonstrated the efficacy of CRT in the treatment of vulvar cancer [15], [16], [17], [18]. A number of retrospective studies have examined the tolerability of and outcomes after chemoradiation therapy in patients with primary vaginal cancer, but none have had a comparative radiation-only group. Dalrymple and colleagues reported on a retrospective series of 14 patients with squamous carcinoma of the vagina treated with CRT consisting of 5-FU, cisplatin or mitomycin-C; 9 patients were alive and cancer-free 74–168 months after treatment [12]. Samant and colleagues reported a similar retrospective series of 12 patients with primary vaginal cancer treated with CRT, with a more homogeneous treatment regimen consisting of concurrent weekly cisplatin [13]. The 5-year overall, progression-free, and locoregional-progression-free survival rates were 66%, 75%, and 92%, respectively, in that series. Nashiro and colleagues reported on a smaller retrospective series of 6 patients treated with cisplatin-based CRT, of whom 4 remained free of disease at 18, 23, 33 and 55 months, respectively. [14] Our current study, with 51 RT-only and 20 CRT patients, represents the largest series of chemoradiation compared to radiation only for patients with primary vaginal cancer treated at the same institution.

The management of vaginal cancer has changed in several ways over the course of the study. Patients treated before 2002 received LDR brachytherapy, whereas those treated from 2002 forward received HDR brachytherapy. Prospective randomized trials in cervical cancer have shown no difference in outcome between LDR and HDR [19]. In addition, the advent of 3-D imaging since the 1980s has enabled more accurate staging of vaginal cancer and improved delineation of the tumor. In order to assess the impact of 3-D diagnostic imaging, this variable was assessed as a potential confounder. In a multivariate model, diagnostic imaging was not significant, whereas CRT remained significant in the final model, indicating the independent predictive role of the use of CRT. The introduction of 3-D imaging has also allowed the implementation of image-guided adaptive brachytherapy approaches over the past decade [2]. Since 2002, our institution has used MRI guidance followed by MR- and CT-based treatment planning for vaginal brachytherapy treatment [20], [21]. In the current study, the prescription dose was 74.4+/−9.3 Gy EQD2 for patients treated with EBRT and brachytherapy. The question of whether patients treated with an adequate dose of radiation, using image-based conformal brachytherapy planning, benefit from concurrent chemotherapy must be assessed in larger clinical trials. Chemotherapy does cause rapid regression of the tumor, resulting in treatment of a small radiation volume. This smaller tumor volume may reduce the amount of rectal tissue exposed to radiation, and the D2cc (dose to 2cc of rectum) predicts for the risk of future rectal morbidity [22].

A previous study using RT alone for vaginal cancer demonstrated excellent 5-year DFS rates of 82% for tumors <4 cm and 60% for tumors >4 cm [6]. It also demonstrated stage-dependent locoregional relapse rates of 68% for Stages I–II and 83% for Stages III–IVA [6]. Our study showed a 3-year DFS rate of 73% for all patients receiving CRT.

Limitations of our study include its retrospective nature, its scope as a single institutional experience, and its inclusion of patients who were treated over a long period. The year of diagnosis was therefore included in the analysis to account for shifts in technology and treatments over time, some of which are known confounders and others that may be unknown. Therefore, appropriate adjustment for year of diagnosis in the multivariate modeling was valuable.

Our data suggest that chemoradiation therapy may be considered in the treatment of patients with primary vaginal cancer. Adequate radiation dosing with the combination of external beam and brachytherapy is critical to the curative management of patients. Though accrual may require international collaboration, prospective trials may be warranted to more thoroughly assess the benefit of adding concurrent chemotherapy to radiation therapy in these patients.
